
JOURNAL INFORMATION
==============================
NLM Title Abbreviation: J Can Health Libr Assoc
Journal ID: J Can Health Libr Assoc
NLM Journal ID: 101229936
Journal ID: JCHLA

The Journal of the Canadian Health Libraries Association

EISSN: 1708-6892
Publisher: Canadian Health Libraries Association / Association des bibliotèques de la santé du Canada

ARTICLE INFORMATION
==============================
PMCID: PMC12352437
DOI: 10.29173/jchla29893
Article ID: JCHLA-46-063
Article version: 1
Subjects: Abstracts

CHLA 2025 CONFERENCE INTERACTIVE WORKSHOPS / ABSC CONGRÈS 2025 ATELIERS INTERACTIFS

Electronic publication date: 2025 Aug 1
Publication date: 2025 Aug
Volume: 46
Issue: 2
First page: 63
Last page: 65
Copyright: © Fournier
Copyright year: 2025
License: This article is distributed under a Creative Commons Attribution License: https://creativecommons.org/licenses/by/4.0/
License URL: https://creativecommons.org/licenses/by/4.0/

==============================

## IW1. Getting comfortable with context: an introductory qualitative research methods workshop

Robin Parker[1], Lindsey Sikora[2], Catherine Giroux[3]
[1]Dalhousie University, [2]University of Ottawa, [3]McGill University

**Topic:** Using examples from their own doctoral research, the workshop leaders will walk participants through the basics of qualitative research methods. This introductory session will help participants get comfortable with the key parts of qualitative research design, such as aligning the purpose, philosophy of science, research questions, methodology, and the methods for collecting and analyzing data. With hands-on activities in small and large groups, participants will explore how to rigorously design and report qualitative research. For librarians and health sciences educators more familiar with the methods commonly used in knowledge synthesis and quantitative research, the purpose and assumptions of qualitative research in health and social settings might feel a bit unfamiliar. This workshop will introduce alternative ways of exploring research questions, focusing on understanding what's happening in specific situations or contexts. Don't know what "philosophy of science" means for research methods? No problem! The workshop leaders will break it down in an easy-to-understand way and introduce some common research philosophies and paradigms. Drawing from their own research experiences, the instructors will share some challenges of trying out new research methods and the possibilities that qualitative research opens up to critically dig into the issues of an increasingly complex world. This workshop offers a chance to broaden your research toolkit or explore a new approach for your next research project. **Objectives:** To develop an understanding of the essential elements of qualitative research design and enhance confidence in supporting or conducting qualitative research.

## IW2. Designing instructional strategies for teaching effective use of AI search engines

A key role of librarians who teach is to guide learners in selecting the appropriate tool for the task at hand and using it effectively. With the recent proliferation of AI search engines (eg. Consensus, Elicit, Perplexity, etc.), there is an opportunity for librarians to harness what we already know about teaching information resources to help our learners integrate the use of these tools into their research practices in a way that's effective and thoughtful. During this workshop, designed for health information professionals involved in teaching, participants will learn how to create activities that develop their learners' awareness and critical thinking regarding the use of AI search engines. This workshop will focus on instructional design and lesson planning, structured around three key pillars: situational factors, learning outcomes, and active learning. The facilitators will lead participants through a variety of activities, including mind-mapping exercises and group discussions, and will draw on the collective experience of participants as well as their own experience teaching AI search engines. This workshop will inspire participants to collectively explore and reflect on how to incorporate AI search engine instruction into their teaching.

## IW3. Designing resilient library services: a workshop on resilience engineering for librarians

Lorri Zipperer[1], Amanda Ross-White[2], Nicole Capdarest-Arest[1]
[1]University of California Davis, [2]Queen's University

**Topic:** Resilience engineering (RE) offers a powerful framework for designing processes that enable organizations to respond effectively to and learn from large-scale disruptions such as system outages, funding loss, natural disasters, and unexpected leadership transitions. While resilience is often viewed as a personal trait, resilience takes on new significance in RE's systems approach, emphasizing the critical need for organizational-level resilience. This perspective is particularly relevant to libraries, whose operations and services are pivotal to institutional adaptability and sustainability. This 90-minute interactive workshop will introduce participants to the principles of RE, exploring how its application can empower librarians to strengthen organizational resilience. Through real-world examples and participatory activities, attendees will gain practical strategies for integrating RE concepts into their daily work, highlighting how information work serves as a foundation for bolstering resilience at both the library and organizational levels. **Summary of Workshop Objectives:** Participants will leave this workshop with the knowledge to apply resilience engineering principles to enhance library services, develop strategies for building organizational resilience, and advocate for the evolving role of librarians in times of disruption. **Short Description of Interactivity:** Participants will engage in hands-on, practical case studies designed to help them translate RE concepts into realistic scenarios, applying the concept to library services and broader organizational functions.

## IW4. Contextualizing generative AI tools and academic integrity in health research literacy

Rina Garcia Chua, Talia Greene, Jane Jun
University of British Columbia Okanagan

Generative AI Tools are emerging and evolving at lightning speed. Many librarians are now compelled or expected to have a baseline knowledge of how these tools work and receive requests for advice on which tools to use in specific contexts. A thorough understanding of the possibilities, risks, and limitations of these tools will allow librarians and researchers to make informed decisions about when and how to ethically and appropriately use and/or endorse these tools. **Activity and Methods:** This workshop will allow participants to critically approach multiple tools and methods of use. Presenters will introduce strategies for effective prompt design and participants will be given the opportunity to apply and adapt the strategies to the selected tools, such as ChatGPT and Elicit. Through exploring and testing these strategies, participants will build their confidence in approaching and develop a stronger understanding of effective uses for GenAI, as well as inappropriate applications for AI use. **Goals and Objectives:** The goal of this workshop is to gather a baseline critical understanding when approaching GenAI tools with academic integrity, learning the basics of prompt design/engineering, and applying the knowledge and critical analysis to potential cases in which you may use or recommend the tools as librarians. Additional topics covered include Copyright, Privacy Impact Assessments (PIAs), licensing considerations, and other risks.

## IW5. Data for decision making: what data do information professionals need for effective service delivery, advocacy, planning and professional development?

Minakshi Sharma[1], Tara Landry[2], Marie-Helene Nicol[2], Alexandre Amar-Zifkin[2]
[1]Toronto Public Health, [2]Université de Montréal

The CHLA/ABSC strategic plan has been a useful guidepost for our association's activities from 2023-2025. As the association and its members consider the priorities for the coming years, we would benefit from reflecting on and learning about ourselves. The collection and use of qualitative and quantitative data from members (about themselves and their parent organizations) would support advocacy, planning and professional advancement. We propose an interactive activity where members can engage in scenario or problem-based discussions that highlight the value of data in making decisions. **Goal:** To discuss what type of qualitative or quantitative data could assist in planning, advocacy or professional development. **Format:** Small group. discussion in Home Groups and Expert Groups to generate ideas for CHLA/ABSC members to consider. **Overview:** The facilitators will plan focused discussions to capture input on: (1) what data may be useful to members, (2) availability of data (is it already available? If not, how could we collect it?), and (3) why it may be useful for CHLA/ABSC and its institutional or individual members to have access to data.

JOURNAL INFORMATION
==============================
NLM Title Abbreviation: J Can Health Libr Assoc
Journal ID: J Can Health Libr Assoc
NLM Journal ID: 101229936
Journal ID: JCHLA

The Journal of the Canadian Health Libraries Association

EISSN: 1708-6892
Publisher: Canadian Health Libraries Association / Association des bibliotèques de la santé du Canada

ARTICLE INFORMATION
==============================
Subjects: IW = Interactive Workshops

IW1. Getting comfortable with context: an introductory qualitative research methods workshop

Parker Robin 1
Sikora Lindsey 2
Giroux Catherine 3
1 Dalhousie University,
2 University of Ottawa,
3 McGill University

JOURNAL INFORMATION
==============================
NLM Title Abbreviation: J Can Health Libr Assoc
Journal ID: J Can Health Libr Assoc
NLM Journal ID: 101229936
Journal ID: JCHLA

The Journal of the Canadian Health Libraries Association

EISSN: 1708-6892
Publisher: Canadian Health Libraries Association / Association des bibliotèques de la santé du Canada

ARTICLE INFORMATION
==============================
Subjects: IW = Interactive Workshops

IW2. Designing instructional strategies for teaching effective use of AI search engines

Fuller Kaitlin 1
Nekolaichuk Erica 2
1 St. Francis Xavier University,
2 University of Toronto

JOURNAL INFORMATION
==============================
NLM Title Abbreviation: J Can Health Libr Assoc
Journal ID: J Can Health Libr Assoc
NLM Journal ID: 101229936
Journal ID: JCHLA

The Journal of the Canadian Health Libraries Association

EISSN: 1708-6892
Publisher: Canadian Health Libraries Association / Association des bibliotèques de la santé du Canada

ARTICLE INFORMATION
==============================
Subjects: IW = Interactive Workshops

IW3. Designing resilient library services: a workshop on resilience engineering for librarians

Zipperer Lorri 1
Ross-White Amanda 2
Capdarest-Arest Nicole 1
1 University of California Davis,
2 Queen's University

JOURNAL INFORMATION
==============================
NLM Title Abbreviation: J Can Health Libr Assoc
Journal ID: J Can Health Libr Assoc
NLM Journal ID: 101229936
Journal ID: JCHLA

The Journal of the Canadian Health Libraries Association

EISSN: 1708-6892
Publisher: Canadian Health Libraries Association / Association des bibliotèques de la santé du Canada

ARTICLE INFORMATION
==============================
Subjects: IW = Interactive Workshops

IW4. Contextualizing generative AI tools and academic integrity in health research literacy

Chua Rina Garcia
Greene Talia
Jun Jane
University of British Columbia Okanagan

JOURNAL INFORMATION
==============================
NLM Title Abbreviation: J Can Health Libr Assoc
Journal ID: J Can Health Libr Assoc
NLM Journal ID: 101229936
Journal ID: JCHLA

The Journal of the Canadian Health Libraries Association

EISSN: 1708-6892
Publisher: Canadian Health Libraries Association / Association des bibliotèques de la santé du Canada

ARTICLE INFORMATION
==============================
Subjects: IW = Interactive Workshops

IW5. Data for decision making: what data do information professionals need for effective service delivery, advocacy, planning and professional development?

Sharma Minakshi 1
Landry Tara 2
Nicol Marie-Helene 2
Amar-Zifkin Alexandre 2
1 Toronto Public Health,
2 Université de Montréal

